# Utilisation of New Zealand Rugby's concussion management pathway: A mixed methods investigation

**DOI:** 10.1002/ejsc.12213

**Published:** 2024-11-05

**Authors:** Danielle M. Salmon, Marelise Badenhorst, Sierra Keung, Zachary Yukio Kerr, Johna K. Register‐Mihalik, Janelle Romanchuk, S. John Sullivan, Christina Sutherland, Chris Whatman, Simon R. Walters

**Affiliations:** ^1^ New Zealand Rugby Wellington New Zealand; ^2^ School of Sport and Recreation Sports Performance Research Institute New Zealand Auckland University of Technology Auckland New Zealand; ^3^ Department of Exercise and Sport Science Matthew Gfeller Sport‐Related Traumatic Brain Injury Research Center University of North Carolina at Chapel Hill Chapel Hill North Carolina USA; ^4^ Department of Exercise and Sport Science University of North Carolina at Chapel Hill Chapel Hill North Carolina USA

**Keywords:** communication, community perceptions, concussion, delivery of healthcare

## Abstract

Concerns around concussion highlight the need for strategies to improve the implementation and translation of concussion guidelines in community sports. This study assessed players' utilisation and compliance with New Zealand Rugby's Concussion Management Pathway (CMP). This pragmatic, mixed methods study comprised of concussion injury surveillance and mapping of players' healthcare touchpoints through the CMP. Semi‐structured interviews were conducted to understand stakeholders' experiences. Over the season, 27 different healthcare pathways were identified with 28% of players progressing through all phases of the CMP (ideal pathway). Of the 206 suspected concussions reported over the season, 70% were logged in the CMP phone App, and 89% of these had an associated valid baseline concussion assessment. Prior to returning to contact training, 64% of players obtained a medical clearance. One theme, ‘belief in the value of pathway’ was identified as a facilitator across all CMP components. Themes such as ‘concussion knowledge and the nature of concussion’; ‘communication between stakeholders and sufficient information on the process’; and ‘strong relationships and clarity around responsibilities’ were identified as facilitators across several pathway components. Other facilitators included ‘ease and timing of general practitioner access’; ‘the phone App as facilitator to logging’; and ‘spotting for concussion as a team’. Additionally, the findings signify baseline testing as a potential pre‐cursor to overall CMP compliance. Strategies that support the facilitators identified in this study may further enhance compliance.

## Highlights

- Many barriers to optimal concussion management exist at the community level rugby. In response, New Zealand Rugby developed and implemented a community concussion management pathway (CMP) to support improved recognition, diagnosis and management of concussion.
- Not all concussion management barriers are easily surmountable; nonetheless, many success stories also exist. Purposefully identifying and setting strategies in place that support and cultivate facilitators to optimal CMP use may assist in more players following the ideal pathway.
- The study found that stakeholders who prioritise player welfare and other facilitators like concussion knowledge, communication, strong relationships, role clarity and players attending a baseline assessment contributed to creating a successful journey through the CMP.

## 1 INTRODUCTION

Sports‐related concussions and subsequent issues around brain health are a serious concern (Malcolm et al., 2021; Piedade et al., 2021). Although the amount of concussion‐related research is rapidly increasing, uncertainties remain regarding many aspects, particularly in relation to potential long‐term consequences (Patricios et al., 2023). As a high‐paced contact sport, rugby union (rugby) has a relatively high risk of concussion (Prien et al., 2018). To enhance player safety and welfare, World Rugby developed concussion management and pitch‐side care guidelines (World Rugby, 2017). However, the implementation of these guidelines within the community sports setting can be complex and challenging (Clacy et al., 2019; Gardner et al., 2014).

One challenge includes limited awareness of and compliance to sports‐related concussion guidelines by various stakeholders, including general practitioners (GPs), coaches, parents and players (Donaldson et al., 2016). These concerns highlight the need to continue to find ways to improve the multi‐level implementation and real‐world translation of concussion guidelines in community sports (Donaldson et al., 2016; Finch et al., 2013). In some countries, the implementation of concussion protocols (e.g., the National Collegiate Athletic Association Concussion Safety Protocol or the Canadian Guideline on Concussions in Sport) have led to improvements in management and safety of players/athletes (Damji et al., 2018; Macartney et al., 2019; McCrea et al., 2020; Parachute, 2021). To facilitate the translation of World Rugby's guidelines for the New Zealand setting, New Zealand Rugby (NZR) developed and implemented a community concussion management pathway (CMP) to support improved recognition, diagnosis and management through a collaborative effort involving community rugby stakeholders and the use of technology (Salmon et al., 2020a). In 2019, the CMP was trialled in three provincial unions (organisational structures that work with NZR to manage and provide services to clubs and schools in their region.) in NZ.

The CMP was developed using a systems thinking approach which identified the various levels of influence and the stakeholders involved in each of these levels, who could play a critical role in the success of the programme (Clacy et al., 2017). Concussion management is influenced by interactions between multiple stakeholders and factors across the system's hierarchy, including policy, guidelines, culture, training and equipment (Clacy et al., 2017). To ensure a robust evaluation of the CMP, it was essential to document stakeholders' engagement with the system and the healthcare touchpoints each player had through their recovery journey. However, to fully understand how each stakeholder engaged and experienced the CMP, the use of qualitative methodology was critical to gain insight and to understand how they navigated their concussion journey (Donaldson et al., 2012, 2016).

Apart from a systems thinking approach, we also adopted an appreciative inquiry lens to assist us in understanding how favourable changes within a complex sport system may be created (Cooperrider et al., 1995). As a strengths‐based approach, appreciative inquiry focuses on gathering success stories within a system. Identifying facilitators present within these success stories can then be used to initiate change and developed strategies for the future (Richer et al., 2010). With this approach, we aim to identify facilitators to the ideal pathway, which would inform the future rollout of the CMP. This is important in the context of this study which includes the intersection of healthcare, schools and clubs, where access to resources is often limited across all three environments (Lim et al., 2023). We acknowledge that many barriers exist in community interventions such as the CMP. However, many of these barriers, such as the number of available GPs or resources of clubs and schools, are often beyond the scope of the governing bodies of the sport to resolve. Instead, for this study, we chose to focus on the potential within these settings to optimally manage concussions. Therefore, this study aimed to assess the utilisation of and compliance with the various stages of NZR's CMP. A secondary aim was to expand upon these findings by describing end‐users' perceptions of the factors that facilitated optimal pathway utilisation.

## 2 MATERIALS AND METHODS

### 2.1 Design

This study represents one component of a broader programme of work implementing the NZR Community Concussion Initiative (Salmon et al., 2020a, 2020b). A pragmatic, a sequential mixed methods study (Feilzer, 2010; Johnson et al., 2004) was conducted during the 2019 rugby season comprising quantitative CMP outcomes, followed by interviews to understand stakeholders' experiences of the CMP. The focus of pragmatism as a research paradigm is to find the optimal way to provide practical solutions to real‐world problems (Feilzer, 2010; Johnson et al., 2004). A mixed methods approach allows the incorporation of insights provided by both qualitative and quantitative research into developing these solutions (Johnson et al., 2004).

The first phase of the CMP consists of pre‐season baseline testing using the NZR Concussion Assessment Tool (NZRCAT) which was adapted from the Sports Concussion Assessment Tool family (SCAT 3, SCAT 5, Child SCAT) (Salmon et al., 2020c). Suspected player concussions are logged on the NZR concussion management application (App) by a designated person such as the team manager or physiotherapist. The App generates automated email notifications that are sent to the player, their parents/caregivers, coaches, school/club and provincial union. When the player attends a concussion diagnosis assessment, the GP can access the player's baseline NZRCAT results through a web‐based concussion portal, using a unique identifier code contained within the email notification, which is shared by the player or parent. Once a diagnosis is made, the player is then required to complete the NZR stand‐down period (SDP) and follow graduated return‐to‐play (GRTP) and return‐to‐learning (GRTL) protocols. NZR's SDP is 23 days for players under 19 (U19) and 21 days for players aged 19 and over (19+).

After completion of the SDP, the player is asked to obtain medical clearance from the GP before returning to contact training. At the medical clearance assessment, the GP uses the same code to access the concussion portal and determines whether the player is cleared or not to return to contact. Automated notifications are then generated to key stakeholders indicating whether or not the player has been cleared, streamlining the communication process (Salmon et al., 2020a).

**FIGURE 1:**
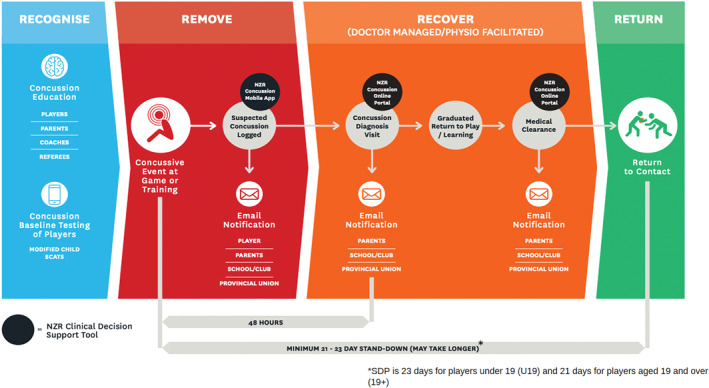
Visual representation of the four stages of the CMP. CMP, concussion management pathway; NZR, New Zealand Rugby.

In stage one of this study, we quantitatively describe players' journeys across the four components of the CMP: recognise, remove, recover and return (Figure 1). In stage two, interviews explored stakeholders' CMP experience. This qualitative data was mapped according to the pathways identified in stage one, to expand upon, and gain a deeper understanding of the CMP (Feilzer, 2010). Approval to conduct the study was granted by (*blinded*) the human research ethics committee (*blinded*).

### 2.2 Sampling and recruitment

**FIGURE 2:**
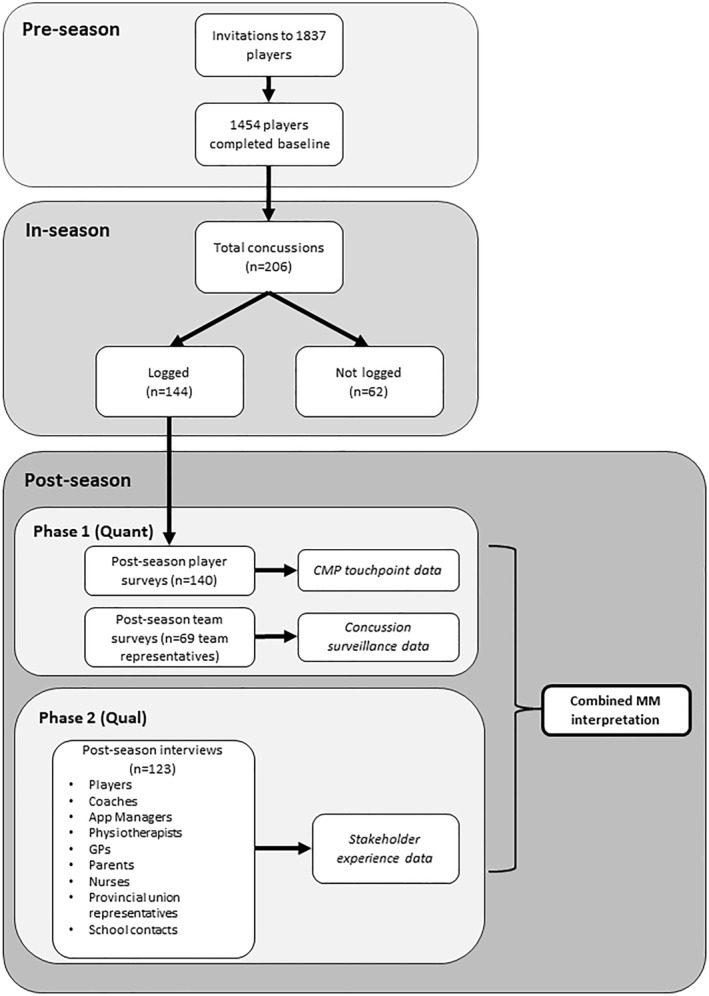
Study design and data collection. CMP, concussion management pathway; GP, general practitioner.

This project was conducted in three geographically and socioeconomically diverse regions in NZ and locality consent was granted from participating provincial unions. Within each of the three participating provincial unions, rugby administrators in schools and premier‐level community clubs were then contacted to identify high schools and clubs that would be interested in participating. During the 2019 pre‐season, a member of the research team attended a training session where players were briefed about the study aims and invited to participate (Figure 2).

A purposive sample of players who had sustained a concussion during the 2019 season as well as other key stakeholder groups were invited to take part in the qualitative post‐season stage 2 of the study. A detailed account of the methods is described elsewhere (Salmon et al., 2024). Invited stakeholder groups included physiotherapists, GPs, team App managers (people responsible for reporting suspected concussions), school contacts, coaches, parents and nurses who had participated at some level in the CMP.

### 2.3 Data collection

#### 2.3.1 Stage 1 (quantitative): Players' journeys through the CMP

##### Concussion injury surveillance data

Post‐season concussion health care touchpoints were exported from the NZR App including the number of players: baselined, concussions logged, medical diagnosis and clearance assessments. In addition to this data research, assistants contacted the respective teams and captured season start and end dates, number of pre‐season and competition games and training sessions per week and any concussion‐related data that was not logged in the App (post‐season team survey). To document the *overall* number of concussions that occurred, teams were asked to report the total number of suspected concussions (logged + un‐logged concussions). This was then compared against the number of suspected concussions actually logged through the CMP App. The demographic information of players whose concussions were logged is contained in Supporting Information S1: Appendix 1.

##### Mapping CMP touchpoints

A unique player ID was used to link all data from the NZR App, mapping players' touchpoints throughout their concussion recovery. If the concussion was not logged, they were contacted post‐season by a member of the research team to retrospective determine what touchpoints each player underwent. Players were asked the following questions relating to the following key outcomes:

1. At the time of injury, did they attend a medical assessment in an urgent care/emergency department?
2. Did they attend a medical diagnosis assessment with a GP?
3. Were they medically cleared before returning to contact training?

All players post‐season were asked the following:

1. Did they follow the GRTP/L guidelines?
2. When did they return to contact training?

#### 2.3.2 Stage 2 (qualitative): Stakeholders' perspectives of players' journeys through the CMP

Post‐season, semi‐structured interviews were conducted to explore stakeholders' perceptions of the CMP. Participants were contacted via phone or email and informed of the goals of the study. A total of 123 participants were included in the study (Supporting Information S1: Appendix 1). Interview questions explored participants' experiences of the phases of the CMP. Additional information regarding the methods is contained in Supporting Information S2: Appendix 2.

### 2.4 Analysis

#### 2.4.1 Quantitative analysis: Deriving concussion prevalence and incidence data

##### Incidence

The incidence of concussion was calculated per 1000 athletic exposures (AEs) and per 1000 match hours. One unit of AE was defined as one game or one training session. The total AE for each team was defined as the number of baselined players in a team multiplied by the total number of AEs. Detailed descriptions of the analysis are contained in Supporting Information S2: Appendix 2.

##### Mapping of CMP touchpoints

Player pathways through the CMP evolved by constructing code‐strings representing the player's journey. First, all player outcomes were assigned a code (e.g. BL = completed baseline, L = concussion logged, GPS = player saw a GP on the CMP system, ED = emergency department, NRTP = did not return to play, RTP = return to play and MC = medically cleared). Second, player pathways were constructed according to the codes to depict the player's journey through the CMP (e.g., BL‐ED‐NRTP) and assigned a number that represents that specific pathway (e.g., Pathway #1: BL‐L‐GP‐MC‐RTP). Pathways were then aggregated across players to determine the frequency of the pathways across all players who sustained a concussion.

#### 2.4.2 Qualitative analysis: Interview data and integration of quantitative data

Interview recordings were transcribed verbatim and organised in NVivo 12 (QSR International). A framework analysis approach was used to analyse all qualitative data, providing a structure into which data could be systematically reduced to facilitate analysis and comparisons across stakeholders (Gale et al., 2013). During this process, weekly meetings were held with the core coding team (MB and CS) to discuss coding and test assumptions. The broader research team discussed the coding process and provided input on a bi‐weekly basis. Once coding was completed, one researcher developed multiple framework matrices by summarising the data by category from each stakeholder group according to each phase of the CMP. The content of the matrices was discussed and revised as part of weekly team meetings during the analysis phase. The matrices provided a structured overview of summarised data and facilitated the recognition of patterns and themes across the dataset (Gale et al., 2013).

For the purpose of this study, the qualitative analysis adopted an appreciative inquiry lens and was focused on data related to the main quantitative objectives from stage 1. The main themes around participants' perceptions of facilitators related to each section of the pathway were tabulated. An iterative process considering how the qualitative data expands upon or illuminates the quantitative data was undertaken. The final goal of the analysis was to integrate the quantitative data describing the utilisation of the CMP with a qualitative account of the underlying process to illuminate potential facilitators to optimise the utilisation of the pathway (Moseholm et al., 2017).

## 3 RESULTS

Results are presented by first describing the overall pathways utilised and the main themes identified by the qualitative analysis. Thereafter, quantitative and qualitative results are presented for individual pathway components including (i) baseline testing, (ii) logging of the concussion (with the inclusion of incidence results), (iii) diagnosis visit, (iv) medical clearance visit and (v) completion of SDP/return to play/learn guidelines.

### 3.1 Mapping of CMP touchpoints—Overall pathways utilised

**FIGURE 3:**
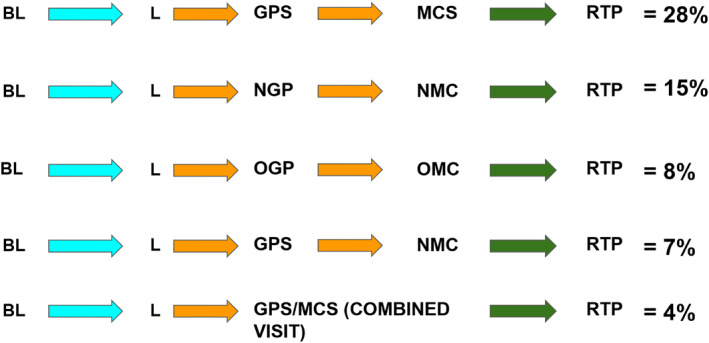
Five most common pathways utilised in the NZR CMP (% = frequency of players). BL, Baseline completed; CMP, concussion management pathway; GP, general practitioner; GPS, Player saw a GP on CMP system; L, Concussion logged; MCS, Medically cleared by GP in the system; NGP, Player did not see any GP; NMC, No medical clearance obtained; OGP, Player saw own GP; OMC, Player saw own GP for medical clearance; RTP, Returned to play.

Players undertook 27 different pathways following a suspected concussion. The five most common pathways are presented in Figure 3. The most utilised pathway was BL‐L‐GPS‐MCS‐RTP, which was also the ideal/intended pathway.

**FIGURE 4:**
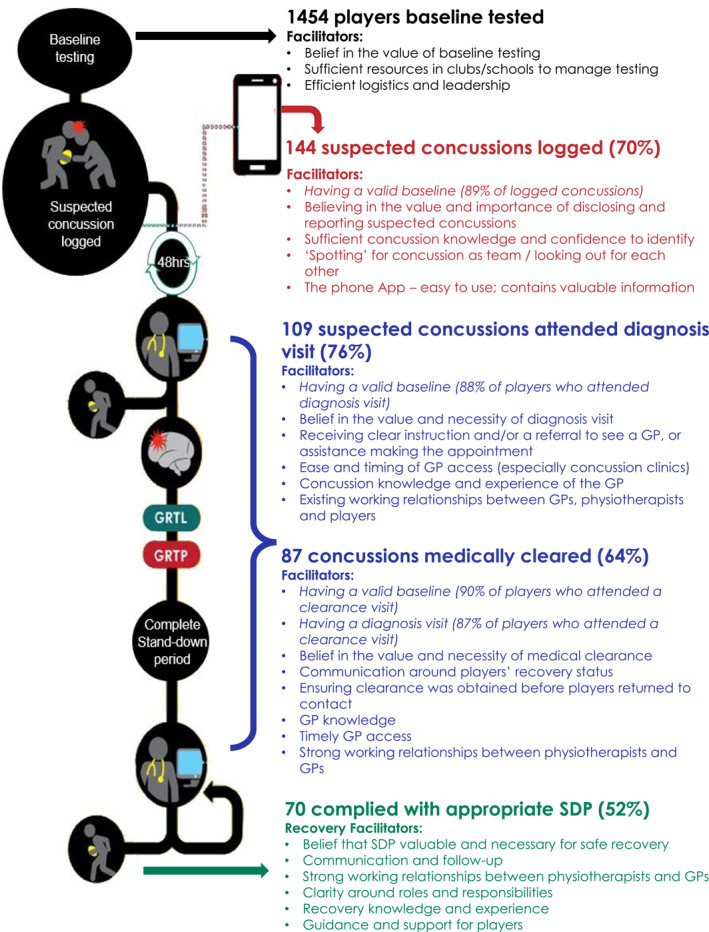
Main facilitators to pathway compliance. Bullet points in italics = quantitative facilitators; non‐italic = qualitative facilitators. SDP, stand down period.

From the qualitative analysis, 10 themes were identified as main facilitators for the optimal pathway flow (pathway 1). For these 10 themes, illustrative quotes are provided according to the relevant pathway component in Table 1. One theme, ‘belief in the value of pathway and diligence in its execution’ was identified as a facilitator across all components of the pathway (Figure 4). Themes such as ‘concussion knowledge and the nature of concussion’; ‘communication between stakeholders and sufficient information on the process’; and ‘strong working relationships, clarity in roles, responsibilities, and authority’ were identified across several pathway components. The following section presents the results related to each pathway component.

**TABLE 1:** Key facilitators for optimal pathway: Pathway 1.

Theme	Theme according to relevant pathway component	Illustrative quotes
1. Belief in the value of the pathway; support and diligence in its execution	1.1) Baseline testing	*I think having the baseline testing was really good, and having a group of us doing it, and even bits of our management team doing it was actually good because they've got kind of an insight into what we're up to.* (C11 Physio)
		*Getting the baseline testing and knowing that was there, it made me more comfortable knowing that if they were going to a GP, it wasn't a, “Oh yeah, played rugby, had a head knock, you've got a concussion.” There was actually some basis behind their diagnosis, because I know they've got short visit times and if they can quickly flick someone through, they probably will.* (C10 Physio)
	1.2) Concussion logged	*I'm quite lucky that everyone got my back. And so, my words were final. Yeah, I didn't really have any pressure and people saying, “Oh, hang on a sec. Just let them keep going or what not.” Yeah, I was pretty lucky.* (B16 Physio)
		*Making sure that we, first of all, know about this and to be honest, we always, we just pull boys and then let the medicals professionals deal with them so obviously (1st XV Team Physio) then, she's our first port of call in terms of that, she's just making the decision and we just don't even argue[…] like we would just not put boys out like that, regardless of whether they're our best player or not, it doesn't matter.* (C9 coach)
	1.3) Diagnosis visit	*I think compared to previous, the awareness is much higher, so people come in and they're ready to not play and ready to understand that they need to look after themselves and they've seen role models doing similar things.* (C40 GP)
		*The coach is really good at making sure they follow through (going to GP) and do that sort of thing. I think our players are quite good there.* (B18 Physio)
	1.4) Medical clearance	*No, I think they need it (referring to medical clearance). Their parents need that medical clearance. They need someone professional, not just me telling them that their kid's good to go. And I think that makes them feel, the doctors told me I'm better. So yeah.* (C32 App manager)
		*I think that was the biggest thing for me as a coach anyway. Peace of mind, yeah. That they'd been cleared.* (C15 Coach)
		*If someone's had a head knock, then it's mandatory that they can't continue to do anything until we received something formally. They have to go to a doctor; we receive a notification from the doctor to clear them. And all managers know that. Yeah, no one gets in until we get the email notification.* (A18 school contact)
	1.5) Return to play—stand down and recovery	*I had to have a break so I kind of … as much as I didn't like it, I did, because it gave me time to recover and stuff like that.* (C2 Player)
		*I guess from our perspective, it seems like the coaches and the whole team management have been really on board and supporting the boys, which is really awesome to see. They were aware that (her son) was really frustrated as well and that he was feeling… It got to a few weeks post‐injury, and he was feeling like he was missing out, and they were aware of that, but they just were really good at reminding him that at the end of the day, it's only a few games and your health's way more important. So, it was quite good that they were relaying that back to him, like, “Just be patient. It's better to get this sorted out. It's no point coming back but only being halfway there.” So that was really good as opposed to them saying, “When you going to get back on the field? Hurry up and get better.” They were much more about saying, “It's okay. Just take your time. It's not the end of the world. It's only a few games.”* (B33 Parent)
		*Our management staff definitely were like real strict on it. And they knew, they wouldn't pressure us to start training if we weren't able to. So even if we tried to convince him when we felt better, he still wouldn't let us, kind of thing.* (A9 player)
2. Communication between stakeholders, including follow‐up and monitoring of players and sufficient information of the process	2.1) Diagnosis visit	*Because of my physio … I didn't really have to do anything, I just had to show up. My physio organized it and then coaches pushed for it as well. I didn't really want to go, but my mum also made me go. She said, “This isn't an option.”* (C2 Player)
		*He (GP) gave me like a recovery sheet and that kind of stuff. He gave me a 2‐page paper, I can remember one was the recovery sheet and the other one was my score's compared to what I have done previously. That was good. Having the time, seeing the timeline so I could know, that's how far. That's long I have to go and come back, so yeah.* (B3 player)
	2.2) Medical clearance visit	*Again, it comes down to the physio and GP communication, but I don't know whether they always should need to have to go back to the GP to be cleared and we don't… I keep mentioning the GP at Student Health. If I've taken them right through their… I'll just outline what they've done for two and a half weeks, and that they've done this running session, they did this, they did this, that there were no symptoms, and he's happy to clear them without seeing them. He'll send an email back to me with their clearance letter to return to contact. The other thing is, he (GP at student health) knows that they pass contacted training before they're allowed to play. I'll still rule someone out, even if they've been cleared if they don't get through contact. Again, it's that trust and communication. Other than that, probably not really.* (C12 Physio)
		*So, most of the time it was pretty good. Like I'd have the boys had just come and say “yeah, I got cleared”. It was just that one guy that I had to chase, like he thought he had an appointment, but he didn't. So, then I had to chase the doctor up and get like … He just wrote me a letter saying “Yeah, he can do contact”.* (C31 Physio)
	2.3) Return to play—stand down and recovery	*I was usually communicated from coaches because the coach was all about communication. He was always asking about players in regard to how they were training and how they were feeling at training because he would go to me and (physio) about every health‐related issue that he thought that we could see.* (A20 Physio)
		*So, early on when some of the boys got diagnosed, obviously we had a good relationship with the rugby coach, and so they were fully aware of where their kids were at…So, we made sure that we are in contact with the consultant, or specialist, or GP that they had seen. So, either have a verbal handover of their symptoms at the time and asked their advice, or saying, “We want to get them back to controlled contact,” which would be the best in outline. In other words, just pads. Not full contact. If they were happy with it, or if they were going back to contact, we'd make sure that they would get cleared before going back. So, just kind of those simple safety ones for us. Provided that these symptoms matched where they were.* (A28 physio)
		*And then Kayne (physio) or Steve (physio) both will email me and advise me what they're up to, what they can do. So, we're not just running around the track or with the [inaudible 00:21:01] going to do, so then we'll contact people so that they can or can't be involved in it.* (B6 coach)
3. Nature of concussion and concussion knowledge	3.1) Concussion logged	*(Player) got a bit of a history…he had some real time off rugby last year, so he's pretty good at knowing what's going on. I think he probably would have said that yesterday. I think he came to the side‐line, in fact, and said, “I'm not good.” He actually walked off the field himself…* (B5 AM)
		*He wasn't unconscious, but clearly got a knock and stayed down for a minute or two. Again, ran over, checked him, he was quite clearly concussed, we asked him the normal questions and he was a little bit dazed. And so again, he was able to walk, picked him up, walked him off the field and sat him down.* (A3 AM)
		*Generally, I recognize a concussion when it happens on the field, and I'll run out and I'll ask a couple of really simple questions and then if I just check them again off the field. I was logging it straight away, giving them, the player, verbal information on the side‐line, go up to (sport medicine concussion clinic in area) and request a concussion appointment.* (A7 Physio)
	3.2) Diagnosis and medical clearance visits	*I think… I mean, you're much more used to doing head injury assessment because you've been doing it for years and at a very high level, and I think depends on if this is something that's going to get rolled out to all GPs, I actually think one of the strengths of this is that it gives that structure for the GP, or whoever's doing the assessment to work through, and I think that's, from my own personal point of view, that was something that I really felt at the end of it there's a really good structure.* (A23 GP)
		*Yeah. So, you found when they came here (Concussion Clinic), they had a really clear process to follow. Whereas maybe their GP doesn't see that many concussions or isn't aware of the SCAT5 or something. Some of them weren't even told… One of them was told, “Oh just don't train tonight and then you'll be fine the next day,” kind of thing. You know, just not great education and… Yeah. Whereas if they came here (Concussion Clinic) they got really good education.* (C18 physiotherapist)
		*I found last season in terms of what I did, it was still pretty much exactly the same. But them [player finding a doctor who actually knew about it was quite hard work. Quite a few people come back with that “Oh well I don't know if he's concussed or not”. Which then made me the bad guy, when I was like, “Well you can't play then”. So having a team of doctors that were sort of concussion based was helpful. Made it a hundred times easier. Yeah.* (C31 physiotherapist)
	3.3) Return to play—stand down and recovery	*If I didn't think they were ready, they didn't do it. Because I've seen them running, because by the time they're doing that I've been running with them for two weeks, or week and a half. If they're getting to each step and they're not having any symptoms all the way through, by the time they get to that point… If they're doing contact on a Thursday, on the Tuesday they've done sprints and they've done down‐ups, and if they've got through that fine, then I'm pretty confident in having them hit a hit shield.* (C12 physiotherapist)
		*I had treated so many concussions or had dealt with so many concussions I usually know what stage they need to be at and that kind of stuff.* (A27 physiotherapist)
		*Yeah, 21 days is a pretty good stand‐down for … but obviously, there's borderline on to a very mild concussion, the ones that just bounce back up straight away after just two days, or maybe just suffering in the acute phase and by the time I see them on Tuesday, no symptoms. And those are the kind of the ones that we need to monitor. It's a hard one because three days after and if it's no symptoms whatsoever then they'll just go and do training and if they do training on Tuesday and they're absolutely fine then we'll just progress it accordingly and just try and do a bit of harder ones one Thursday. They might miss game on that week but next week do a contact training and if it's absolutely fine we'll jus actually go with them.* (A27 physiotherapist)
4. Ease and timing of GP access	4.1) Diagnosis visit	*The concussion clinic was like… I did the test and after that it's straight “Go to the meeting with doctor”. That was kind of good. Like, I can mentally, I don't know how to say this quite right. Mentally satisfied. Like can relax because I was really nervous about my concussion because I had a headache, so yeah, I think after meeting with the doc I was pretty relaxed.* (C43 player)
		*Oh, one thing that was good this year was the potential for the players that aren't students to be able to see a GP here (Concussion Clinic). I don't actually know if we had any of them that weren't students. Like in university we might have maybe five players on our team that aren't studying. Last year we had real issues with them not being able to… If they had been studying for three years, and then they started working, and they weren't at a GP practice, no one would see them. Student Health wouldn't see them. GP practices… There was one where he was charged $80 or $90 or something. It was really tricky sometimes for them to actually see anybody.* (C12 physiotherapist)
	4.2) Medical clearance visit	*Yup. So, I do think that we had that after hours clinic, which I think was really useful for players to be able to get direct access and timely access to who can assess them and clear them.* (C38 GP)
		*I think, because [person working for NZR] sent out a list of all the doctors that we could send to… So that was probably the most helpful part for us, was to make sure that if axis weren't available, who else do we send our kids to. Other than the contacts that we already have. So, it's just, yeah, those things were probably the most helpful.* (A28 physiotherapist)
5. Strong working relationships between stakeholders, clarity in roles, responsibilities and authority	5.1) Diagnosis visit	*Yeah, it (diagnosis visits) was good. I've got a pretty close relationship with the docs, so it's fine.* (B22)
		*Maybe because I know (GP) quite well. I've been rather injury prone in my season, so I think he knew that I knew that I can listen to my body, and I do that well.* (B27)
		*There'll be others who will, who emailed me directly, so that was nice. So that might've been the ones that I might have had relationships with.* (C30)
	5.2) Medical clearance visit	*Yeah, and I think it got easier as the season went on, and as the players were going through concussion clinic and once they'd had that initial appointment, they'd often had a second one clear them for contact, but through that last return‐to‐play week, we could email through to the GP and be like, “Look, this is how they're going, this is what their symptoms are” and get almost a virtual clearance of them. Which saved a lot of time with the clinics not being, you know, every night. So, you know, you want your players to play on the weekend.* (C11 physiotherapist)
		*Probably the only thing is that… Again, it comes down to the physio and GP communication, but I don't know whether they always should need to have to go back to the GP to be cleared and we don't… I keep mentioning the GP at Student Health. If I've taken them right through their… I'll just outline what they've done for two and a half weeks, and that they've done this running session, they did this, they did this, that there were no symptoms, and he's happy to clear them without seeing them. He'll send an email back to me with their clearance letter to return to contact. And again, that's built upon the fact that I've worked with him for a few years, and there's some level of trust that he knows that if I'm saying that they have done this, this and this week and they've had no issues, and I'm happy with where they're at, and he's happy to take my word for that. If I say, even though that they're going really well, they're still not right in regard to this, hes happy to take my word for that as well.* (C12 physiotherapist)
	5.3) Return to play—stand down and recovery	*Yeah, I definitely think, especially doctors like [sport medicine physician], are very, very clear. They send a letter and say this is the plan, this is how many days they're going to do this, this is their heart rate max, this is when they can return to contact. Whereas I don't think other doctors actually did that kind of stuff.* (A7 App manager)
		*So, when I think of our management team, I think of myself (Team Physio), our team Manager, our two coaches, and our strength and conditioning coach as well. So, we all do a Facebook chat of all of us. It keeps everyone on the same page.* (C11 physiotherapist)
		*At the (sport med clinic) system they're quite good at just telling what to do and what not to do and how much they should be doing and that kind of stuff. They're quite good at that part of it.* (A27 physiotherapist)
6. Guidance and support for players	Return to play—stand down and recovery	*Yeah, as I said before, like I, even though I had a rest for three weeks, still had a dizziness, or some neck pain. So, I think that this process will work well…Just because I could talk to the doctor consistently, like, so yeah. I was pretty confident too, to get back in the game.* (C43 player)
		*I think just touching base with them and probably reiterating what the doctor had said, so they know exactly what they can and can't do. The initial stages probably not anything structured that I would do. But yeah, just helping direct them, especially with university work and all that stuff as well. Because a lot of them obviously just focus on rugby, but there's all the rest of life as well, and actually understanding getting back to normal life is first rather than just worrying about rugby.* (C24 physiotherapist)
		*So, in that second time that I visited him. He told me that he wasn't going to clear me and that he wanted me to, not to push at the start. And he gave me dates, so he said, “In four days, I want you to go for a run for at least three kilometres,” I think he said, “and then go home and kind of just reflect on how you feel that next hour or so,” I think is what he said. “If everything there is okay, I want you to go to the gym and have a workout and make sure everything is all right there. And then after then, you can return to training and not do any contact, but you can train and do everything else.” And after all that, I went back, and I chatted with him and I was happy and he was happy. So I could go back to training and do contact and he was happy with it and cleared me.* (A30 player)
7. Sufficient human resources	Baseline testing	*So, they (NZR) helped us resource it, because in reality that was quite a big task. Yeah, and bear in mind we're all volunteers, typically, doing this… And so, in a way, you guys are asking us to give up a fair bit of time for this. And for the most part, I think most of us would be fine with it, I was fine with it. But having one or two people come out to help… turned it from a six‐hour job into a two‐hour job, and that made a massive difference.* (C30 physiotherapist)
		*When we did finally get all sorted out, having the extra hands down to help get through the baselines were good.* (C20 physiotherapist)
8. Efficient logistics, leadership, organisation and timing	Baseline testing	*I think any proactive club would be all over that. A five‐minute baseline test. You've got a pre‐season of two months. So, if you can't get 30 players baselined over a two‐month period, then you've got some problems…. You'll get the ones that aren't proactive and won't do anything.* (A22 Provincial union representative)
		*Doing it at training (went well) because we had the players there. It wasn't out of their normal routine, so it was easy enough just to pull players in and out and get it all done. Most of the bulk of the player's done quickly. That was good. It was good practice for me as well, going through the testing.* (C24 physiotherapist)
9. App as a facilitator to logging	Concussion logged	*Knowing that there's something in place too, rather than all that pressure just being on us to think on our feet, and having a tool there. Like there was so many times where I would just go through it. Just the questions in the app, I even actually used it with a different patient in the clinic.* (C10 physio)
		*It was a simple to use app, with everything that you'd want to ask the player at that point. Then you get them sent through all that stuff that needs to happen, then they go to (Auckland based sport medicine clinic) or another sports concussion clinics to get diagnosed and then put through the system as quickly as possible, which I thought was really cool…To get them back into playing as safely as possible….and I guess the App is designed to try and facilitate that return to play as quickly and as safely as possible, so it's beneficial. I think once people buy in, if that becomes a nationwide thing and people just start thinking it's normal, then I think that it's going to be great, so it'll just be easier. Even though more people know about concussions, this App is to streamline the process as fast as possible and then I think everyone will buy into it anyway….I found the App really like quite simple to use, especially if I'm on the field for example, and I'm using especially that button we said and you get them up, you give off those symptoms that in regards to …Red flags…And maybe you do need to ask those questions for example of me being a student, because I might not know much about what are the right questions to ask due to my lack of experience around concussion. I haven't been in that situation enough. I press that button and I ask the players are you feeling any of these, are you feeling any symptoms and then they'll be like yes or no, then that gave me, that made me feel like I was confident enough to be like, you need to go be seen by a medical professional at this point or not….* (A20 physiotherapist)
		*Oh yeah, no, that would… If you weren't aware of those, I think if you were a coach or a family member or something like that, it (the App) is very, very helpful and it was quite handy for me to be able to check…* (A7 physiotherapist)
		*I found, you know, when you're like logging it and it comes up with like all the red flags and stuff. That was really good, because it was just a very easy. Like, I didn't have to remember them I could just read them out and say “Hey if you get any of these, go to the hospital”.* (C31 Physiotherapist)
10. Spotting for concussion as a team	Concussion logged	*Yeah, they said that I wobbled around, and I fell over again. And then I tried to get up and carry on playing and they tried to stop me from trying to carry on playing when I apparently was shouting that I wanted to keep playing. But then they said that they took me off and then I kind of realized that I was a bit, unwell, you know. My mates looked out for me, the coach was there, he was all good with it.* (A14 player)
		*To show you from a team point of view, our captain…got knocked out in a very, very important game for us, between […]. The first round was pretty much a must win game for us. He took a knock. I didn't see it and half time he was a bit dizzy, and it was the vice‐captain (player), came over says he needs to go off.* (B6 coach)
		*We had an incident where one of the younger guys got a head knock. And we couldn't see it from the side‐line, he didn't fall over, he wasn't lying on the ground. He just… We put the ball on the wrong side of the scrum and the captain stood up and looked at him and asked if he feels all right and quite clearly, he wasn't, and (player) grabbed him, brought him to the side‐line and he just said to the ref, “I'm taking (player) off.” So, they were much more aware of what to look for and how dangerous it was. And we wouldn't have seen it, (player) would've played on. He's the captain now, he would have played on had it not been for the other players.* (A5 App manager)

### 3.2 Baseline assessment

A total of 1837 players from 61 clubs and schools were invited to participate, and 79% (*n* = 1454) of players consented and completed a baseline assessment (males *n* = 1273; females *n* = 181). Of the 144 concussions that were logged using the App, 89% (*n* = 128) had valid baselines.

Three main themes were identified as facilitators to obtain a baseline assessment: belief in the value and necessity of baseline testing for concussion management (Table 1; facilitator 1.1); sufficient resources in schools and clubs to manage testing (Table 1; facilitator 7); and efficient logistics and leadership: these included sufficient human resource support to conduct baseline testing, a proactive person (such as a physiotherapist or team manager) who planned and supported the testing and organisation of testing at an appropriate time (Table 1; facilitators 8).

### 3.3 Logging of suspected concussions

Over the 2019 season, in the 3 provincial unions, a total of 206 suspected concussions were reported post‐season, 98 in the premier level (highest level of amateur rugby in NZ) and 108 at the high school level, equating to a combined rate of 12.37 concussions per 1000 player match hours. Of the 206 suspected concussions reported over the season, 70% (*n* = 144) of these were logged in the App for a total of 140 players (four players sustained two concussions over the season). The incidence rate for the logged concussions was 8.47 per 1000 player match hours. The combined exposure rate for total concussions (logged concussions + un‐logged concussions) encompassing games and training was 2.79 suspected concussions per 1000 AEs and 1.91 for logged concussions. The total number of suspected concussions was 8.56 per 1000 AE of match play.

Logging of a suspected concussion was facilitated by favourable concussion attitudes and buy‐in towards the CMP. This buy‐in included belief in the value and importance of disclosing and reporting suspected concussions and stakeholder support for the person logging the concussion, knowing this would set the mandatory SDP in motion (Table 1; facilitator 1.2). Concussion knowledge and the severity of concussion played a role in recognising/identifying the concussion as a precursor to logging (Table 1; facilitator 3.1). Participants felt that players with obvious signs were easier to identify and remove from the field. Nonetheless, coaches' or physiotherapists' concussion knowledge and experience in managing these injuries provided confidence when identifying concussions. Similarly, players who were aware of the signs and symptoms and what to look out for, as well as the protocol that must be followed after a suspected concussion occurred, acted as valuable facilitators.

The recognition of a concussion by others acted as a major facilitator in getting a player removed from the field after a suspected concussion (Table 1; facilitator 10). Having multiple stakeholders during the game alert to potential signs of a suspected concussion was beneficial. These ‘spotters’ included players reporting on behalf of their teammates, coaches, team management, medical staff (physiotherapists), parents and the referee (blue card [Visual cue used by the referee to immediately remove a player from the field if there is a suspicion of concussion.]). The App facilitated the process of logging by being easy to use and containing valuable information, such as what the concussion ‘red flags’ were and what to look out for post‐injury (Table 1; facilitator 9). This gave physiotherapists and App managers confidence to ask the right questions without worrying about remembering all the ‘red flags’ correctly. The App also gave clear guidance on what information should be passed along to the injured athlete and their caregiver to support the initial 48‐h period (i.e. not drive home if red flags seek immediate medical care).

### 3.4 Diagnosis visit

Of the 144 logged concussions, a diagnosis assessment with a GP was sought for 109 (76%). The breakdown of diagnosis touchpoints is the following: for 71 suspected concussions, players saw a GP who had access to NZR's GP portal; in 23 instances, players saw their own GP in six instances and players saw an ED doctor. Nine were cleared of concussion at their ‘diagnosis’ visit (five from a GP within the CMP system and three from their own GP, and one was cleared by ED), and 35 failed to attend a diagnosis assessment. Of the 109 suspected concussions that attended a diagnosis visit, 96 (88%) had valid baselines.

From the qualitative data, five facilitators were identified that supported the attendance of a diagnosis assessment. These included stakeholders' belief in the value and necessity of the diagnosis visit (Table 1; facilitator 1.3) and communication between stakeholders (Table 1; facilitator 2.1). Receiving clear instruction and/or a referral to see a GP or assistance making the appointment appeared to be particularly valuable. Environments or contexts that facilitated easy and timely access to GP appointments and acute care concussion clinics were highlighted as strong facilitators to this process (Table 1; facilitator 4.1).

Concussion knowledge and experience of the GP (having an understanding of the CMP process, sufficient concussion knowledge and experience in diagnosing players with concussion) as well as human resource and operations capacity (e.g. time available for consultations or assistance from other staff) when available positively influenced the diagnosis process (Table 1; facilitator 3.2). Specifically, acute care concussion clinics with GPs experienced in dealing with concussions facilitated the diagnosis of players. Existing working relationships between GPs, physiotherapists and players facilitated a smooth diagnosis visit, paving the way for access and improved communication between healthcare providers (Table 1; facilitator 5.1).

### 3.5 Medical clearance visit

Prior to returning to contact training, medical clearance from a GP was sought in 64% (*n* = 87/135) of suspected concussions (suspected concussions that were cleared (*n* = 9) at the diagnosis assessment have been removed). Of those who obtained a medical clearance, 66 were from a GP who used NZR's GP portal and 21 were by their own GP. Of the 87 suspected concussions for which a medical clearance visit was sought, 78 had valid baselines (90%). For those confirmed concussions that were medically cleared, 87.4% (*n* = 76/87) also obtained a medical diagnosis of a concussion.

Stakeholders' (including those in a supervisory capacity) belief in the value and necessity of medical clearance before players returned to contact was identified as critical to this stage of the CMP (Table 1; facilitator 1.4). The medical clearance process was further facilitated by good communication between stakeholders around players' recovery status and the outcome of the clearance process (Table 1; facilitator 2.2); sufficient GP knowledge around concussion management (Table 1; facilitator 3.2); and timely GP access (Table 1; facilitator 4.2). Strong working relationships between GPs and physiotherapists meant that GPs could clear players remotely based on physiotherapists' feedback and clinical recommendations (Table 1; facilitator 5.2).

### 3.6 Return to play, recovery and compliance with SDP

For logged suspected and subsequently confirmed concussions, 52% complied with the appropriate SDP (*n* = 70/135). In this cohort, *n* = 7 had SDPs which were extended due to symptoms. In contrast, 7% (*n* = 10) RTP too early and a RTP date were not obtained for 35% (*n* = 48) of the suspected concussions. The length of time from logged suspected concussion to medical clearance for return to contact training for the overall sample was on average 21 days (median 19 days; range 7–57 days). For high school players, this was on average 24 days (median 23 days; range 14–54 days). Club players reported slightly lower time frames 19 days (median 18 days; range 7–57 days), which likely reflects the 21‐day SDP compared to the 23‐day SDP for players under the age of 19.

Belief in the value of the SDP played an important role in supporting players' RTP at the appropriate timepoints. Perceived appropriateness of the length of the SDP, believing that although the SDP was tough, it was valuable and necessary for safe recovery. An additional important facilitator to this process was having the necessary support from the player's school, work or team (Table 1; facilitator 1.5). This was also evident in the emphasis participants placed on the importance of erring on the side of caution (the perception that the SDP can even be longer for more serious concussions or younger players). Belief in the value of the SDP also included perceptions that the SDP increased confidence and certainty that players had recovered and felt ready to return and did not feel pressured to RTP before sufficiently being recovered.

Key factors that facilitated the general recovery of the player included communication and follow‐up with the player during the recovery period (Table 1; facilitator 2.3) and stakeholders' concussion recovery knowledge and experience (especially physiotherapists). Participants reported that ‘milder’ concussions usually had no difficulties progressing through the GRTP and most were ready to return after 3 weeks (Table 1; facilitator 3.3). Clarity around roles and responsibilities of stakeholders during a player's recovery and strong working relationships between GP and physiotherapist were also noted as both facilitators and barriers for this CMP step (Table 1; facilitator 5.3). The importance of sufficient guidance and support for players as they progressed through the steps of GRTP/L was identified (Table 1; facilitator 6).

## 4 DISCUSSION

The aim of this study was to use a mixed methods approach to assess the utilisation of and compliance to the various stages of NZR's CMP, and to add depth to these findings, by describing end‐users' perceptions of the factors that facilitated optimal utilisation of the pathway. Of the 206 suspected concussions reported over the season, 70% of these were logged in the App. Of these, 76% attended a diagnosis assessment, 64% obtained medical clearance and 52% complied with an appropriate SDP. Ultimately, ensuring that players are diagnosed and that they received medical clearance is a key goal of the CMP. In total, the ‘ideal pathway’ was utilised in 28% of suspected concussions (complying to all touchpoints), while another 8% similarly complied with all touchpoints, although utilising non‐system‐based GPs. An important aim of this study was to identify factors that facilitated the utilisation of the ideal pathway. One theme, ‘belief in the value of pathway and diligence in its execution’ was identified as a facilitator across all components of the pathway. The importance of this theme identified in this study is similarly reflected in the diffusion of innovations theory. According to the theory, the successful uptake of an intervention is dependent on whether stakeholders believe the intervention to be better than existing practices. The intervention also has to align with their existing values and needs and to be perceived as easy to understand, use, with clearly visible benefits (Finch, 2011; Rogers, 1995).

However, stakeholders' perceptions of the value of the pathway and buy‐in towards its execution are not just based on their approval of the technical (or practical) aspects and utility of the pathway but are influenced by their underlying attitudes and beliefs around concussions. Belief that a concussion is a serious injury, that it is important to err on the side of caution and that nothing should be placed before player welfare was strongly represented within this theme. Appreciative inquiry emphasises the power of human potential when looking towards creating long‐lasting change (Richer et al., 2010). The human potential identified in this theme is a key facilitator that can be built upon for the future of the CMP. Contrasting attitudes would include downplaying the seriousness of concussions and disregarding the protocols to get back to play regardless of injury, barriers previously described as part of our broader project (Salmon, Badenhorst, Walters, et al., 2022). These attitudes do not create a positive foundation for managing concussion as they do not align with stakeholder values or belief in their existing practices of managing concussion (Finch, 2011; Rogers, 1995). To improve the number of players progressing through the ideal pathway, future strategies should consider how we can build on and enhance stakeholders' belief in the value of the CMP. Changing these attitudes and subsequent behaviours will require more than appealing to people's ‘common sense’ or educating the community about the dangers of managing concussions inappropriately (Kelly et al., 2016). Instead, it will require continued consideration and understanding of the social pressures and underlying motivations that drive behaviours such as ‘being tough’ and playing through injury for the sake of the team (Liston et al., 2018; Salmon, Badenhorst, Walters, et al., 2022). Nonetheless, belief in the value of the pathway does not act alone to facilitate the process. Themes such as ‘concussion knowledge’; ‘communication between stakeholders and sufficient information on the process’; and ‘strong working relationships, clarity in roles, responsibilities, and authority’ were identified across several individual pathway components and have similarly been identified as key factors that contribute to overall outcomes in the CMP as part of the broader project (Salmon et al., 2024). Together, these more ‘practical’ facilitators worked together to create a smooth journey through the CMP and reiterate the importance of these aspects being purposefully set in place and maintained.

For this study, the reported concussion rate was 8.47 concussions per 1000 player match hours for logged concussions, similar to the study by Brown et al. that reported 9.3 concussions per 1000 match hours in a student community rugby cohort (Brown et al., 2019) but higher in comparison to previous studies (Leung et al., 2017; Prien et al., 2018). As postulated by Brown et al., these elevated rates could be attributed to World Rugby's (and the CMP's) ‘recognise and remove’ policy whereby suspected concussions are removed and considered as concussed unless a medical assessment by a GP determines otherwise (Brown et al., 2019; Salmon et al., 2020a). However, in this study, not all suspected concussions were logged, resulting in an overall suspected concussion incidence rate of 12.37 per 1000 match hours. Strategies to support the logging of these suspected concussions are required. Important facilitators to logging included the ease of use of the App, as well as the App's ability to provide guidance, information and confidence to log suspected concussions.

However, for a concussion to be logged, it must first be recognised and reported (Salmon et al., 2020a). ‘Spotting for concussion as a team’ meant players looking out for their teammates and reporting suspected concussions. The importance of engaging the team in concussion safety and reporting was similarly noted in other studies (Kroshus et al., 2016). However, players' reporting behaviours, for themselves or others, may be influenced by various factors including their self‐efficacy to communicate a concussion, perceptions of their coach's attitudes towards concussions, beliefs about the outcomes of concussion reporting and their perceptions of the likely reporting behaviour of other players (Milroy et al., 2020). In this sense, strategies aimed at improving players' social environment and relationships within the rugby team may be key in enhancing players' concussion‐reporting behaviours (Baugh, 2020). The knowledge and experience of supervisory members of the team also assisted in the identification of suspected concussions. Although concussion knowledge does not necessarily translate to positive behaviours, without the required knowledge decision‐making around concussion management may be greatly hindered (Bramley et al., 2012; Kay et al., 2021; Kerr et al., 2014).

Most logged concussions, and those players who attended a diagnosis and clearance visit, had valid baselines, which points to the importance of starting the process ‘correctly’ and potentially signifies baseline testing as a pre‐cursor to engaging in the CMP process. Apart from the belief in the value of baseline testing, efficient logistics, leadership, organisation and timing, as well as sufficient human resources to conduct baseline testing acted as key facilitators. These are factors that may be more difficult to influence and build upon, depending on the extent of resources available within the setting. Nonetheless, other studies have similarly shown that the value of baseline testing may extend beyond its clinical role in concussion management by also acting as an inter‐active educational tool to enhance players' concussion safety decisions (Deuschle et al., 2021).

In this study, medical clearance from a GP was obtained in 64% of diagnosed concussions before returning to contact training. A previous study conducted in New Zealand reported that 52% of players made their own decision to return to play and only 22% sought medical clearance (Sye et al., 2006). Typically, in NZ, attending a consultation with a GP incurs a co‐payment, in addition to what is covered by the Accident Compensation Corporation (ACC), NZ's no‐fault insurance provider. As part of our initial pilot work, it became evident that the co‐payment would be a barrier for certain players. To remove this barrier, NZR covered all co‐payment fees for GP assessments. Although improvement is evident, this study identified the diagnosis and medical clearance visits as specific pressure points within the system. The second most common pathway was characterised by players not attending either a diagnosis or clearance visit before returning to play. The attendance of diagnosis and clearance visits was facilitated by easy and timely access to GPs and acute concussion care clinics. Depending on the context, access to GPs may be constrained and concussion clinics may not be available. In this sense, building relationships and strengthening communication with GPs may serve towards developing a plan that would suit the context and its stakeholders. Physiotherapists with a good working relationship with GPs were able to assist GPs in clearing players remotely, based on their feedback and assessment of the player, which may serve to relieve some of the burdens on GPs in resource‐constrained settings (Salmon, Badenhorst, Falvey, et al., 2022; Salmon et al., 2023). Some players saw their own GPs for diagnosis or clearance. Early communication between the player and the physiotherapist/team lead/App manager about seeing a non‐system GP may allow time to ensure the GP is aware of the existing guidelines and protocols in place. Additionally, receiving clear instruction and/or a referral to see a GP or assistance making the appointment appeared to be particularly valuable for players. Formally assigning this responsibility to a team member knowledgeable about the CMP process and identifying GPs who are familiar with the system, whether it be the physiotherapist or a parent or the coach, may serve to assist players and parents in these initial steps.

In this study, compliance with the appropriate SDP was observed in 52% of concussions, while 7% RTP too early. Although belief in the value and importance of the SDP was a key facilitator, guidance and support for players during the entire recovery phase were also important. In this study, the physiotherapist appeared to play an integral role in providing this support and guidance. Other studies have similarly reported the importance of recovery support provided by athletic trainers or physiotherapists (Wayment et al., 2020). However, research has also shown the value of support received from close friends, other athletes who have experienced concussions and parents during concussion recovery (Kita et al., 2020). This continued support to facilitate recovery and compliance to the SDP may be an important factor to explore further. A study conducted in Australian community rugby union players reported that 78% of players did not receive return‐to‐play advice after sustaining a concussion, and for those who did receive advice, all failed to comply with the 3‐week SDP (Hollis et al., 2012). A study that compared stakeholders' perceptions regarding their roles and responsibilities around RTP in South African community club rugby found that although coaches were perceived by others as the stakeholders with the greatest responsibility regarding concussion RTP, only 43% of coaches indicated that they agreed or strongly agreed that they could assist concussed players with RTP (Kraak et al., 2021). Collectively, this points towards the challenges of translation and implementation of SDP regulations. Having a structured management process, such as the CMP, and guidance throughout the recovery phase may be an important aspect of improving compliance. Just over half of players in the CMP were compliant with the SDP versus none in Australian community rugby (Hollis et al., 2012). For the future of the CMP, the extent of support and guidance received by players, and the persons responsible for this support should be further explored, so that these stakeholders may be utilised to their full potential and GRTP/L protocols may be expanded upon accordingly.

### 4.1 Limitations

As part of the analysis phase, we adopted an appreciative inquiry lens that enabled us to focus on the facilitators of the ideal pathway. Future studies should build on these findings and incorporate the next steps of the appreciative inquiry process, which would include the engagement of community stakeholders to envision future possibilities for optimal concussion management and how they can build on what is already working and utilise existing resources for further growth.

A particular strength of the study is the integration of qualitative and quantitative methods to explore the utilisation and perceptions around the CMP, as it provides a depth of knowledge that would be difficult to achieve through either method in isolation (Tashakkori et al., 2015). Nonetheless, challenges are expected when implementing complex interventions within real‐world settings. In this study specifically, as part of the quantitative analysis, missing data made it difficult to draw conclusions on certain aspects, for example, the RTP dates of players. Finally, it must also be considered that the nature and severity of the concussion can influence players' RTP due to factors such as prolonged symptoms. Nonetheless, ensuring that players are baseline tested, attend a diagnosis visit and do not return to play without medical clearance remains key steps in keeping players safe.

## 5 CONCLUSION

This study found that 28% of players successfully progressed through all phases of the CMP. The optimal pathway use was strongly influenced by stakeholders who believed that nothing should be placed before player welfare. Other pathway facilitators, such as concussion knowledge, communication between stakeholders, strong working relationships and role clarity, were identified to work together in creating a successful journey through the CMP. Finally, the majority of logged concussions, and those that attended a diagnosis and clearance visit, had valid baselines, which points to the importance of starting the process ‘correctly’ and potentially signifies baseline testing as a pre‐cursor to overall CMP compliance. While the removal of a suspected concussion, medical diagnosis and clearance and completion of the stand‐down period are all encouraged at the community level, it is not feasible to mandate and monitor the adherence to these touchpoints. These findings reiterate the importance of purposefully setting strategies in place that support and cultivate these facilitators. Stakeholders responsible for the health and safety of players in other settings may consider the facilitators identified in this study and how these facilitators may be cultivated and supported in their own settings.

## AUTHOR CONTRIBUTIONS

Danielle M. Salmon, Simon R. Walters, Marelise Badenhorst and S. John Sullivan were involved in conceptualising the overall study design. Danielle M. Salmon, Marelise Badenhorst and Christina Sutherland were responsible for the analysis of the data. All authors were responsible for the interpretation of the analysis. All authors were involved in editing drafts of the manuscript.

## CONFLICT OF INTEREST STATEMENT

DS, JR and CS were employed by NZR during the conduct of this study.

## CONSENT TO PARTICIPATE

All participants provided written informed consent. Players consented to all steps in the CMP process, including the notifications sent to their parents/caregivers, coaches, school/club and provincial union. Parental consent was obtained for players younger than 16 years.

## Supporting information

Supporting Information S1

Supporting Information S2

## Data Availability

The datasets used and/or analysed during the current study are available from the corresponding author on reasonable request.
