# Supplementary material for: Utilisation of New Zealand Rugby's concussion management pathway: A mixed methods investigation
Source: Eur J Sport Sci. 2024 Nov 5;24(12):1883–902. doi: 10.1002/ejsc.12213 (PMC11621389; doi:10.1002/ejsc.12213)
Supplement: Supplementary file 1 — Supporting Information S1 [file EJSC-24-1883-s001.docx]

**Appendix 1.** Demographic information.

**Table 1.** Sample characteristics for players with logged suspected concussion (n=140)

| **Age** | | **Sex** | | **Level** | |
| --- | --- | --- | --- | --- | --- |
| **Mean (SD)** | **Range** | **Male** | **Female** | **High school** | **Club/ Premier level** |
| 19.5 (4.3) | 13-36 | 129 | 11 | 72 | 68 |

**Table 2.** Sample characteristics for qualitative analysis (n=123)

|  | **N** | **%** | **Age** | | **Sex** | | **Level** | |
| --- | --- | --- | --- | --- | --- | --- | --- | --- |
|  |  |  | Mean (Std dev.) | Range | Male | Female | School | Club/Premier level |
| **App managers** | 6 | 5 | 38.5 (15.4) | 24-57 | 4 | 2 | 6 | - |
| **Coaches** | 13 | 11 | 44.5 (7.5) | 28-55 | 11 | 2 | 7 | 6 |
| **Parents** | 11 | 9 | 50 (5.6) | 43-58 | 1 | 10 | 11 | - |
| **Physiotherapists** | 24 | 20 | 28.1 (7.7) | 22-52 | 13 | 11 | - | - |
| **Players** | 36 | 29 | 19.8 (4.8) | 14-32 | 35 | 1 | 22 | 14 |
| **Provincial Union reps** | 4 | 3 | 40.7 (19.6) | 29-70 | 4 | 0 | - | - |
| **School contacts** | 14 | 11 | 45 (11.1) | 27-60 | 11 | 3 | 14 |  |
| **GPs** | 9 | 8 | 47.5 (12.9) | 27-62 | 7 | 2 | - | - |
| **Nurses** | 6 | 5 | 34.5 (11.4) | 30-61 | 0 | 6 | - | - |
|  |  |  |  |  |  |  |  |  |
| **Total** | 123 |  | 34.5 (14.6) | 14 -70 | 86 (70%) | 37 (30%) | - | - |
