# Supplementary material for: Utilisation of New Zealand Rugby's concussion management pathway: A mixed methods investigation
Source: Eur J Sport Sci. 2024 Nov 5;24(12):1883–902. doi: 10.1002/ejsc.12213 (PMC11621389; doi:10.1002/ejsc.12213)
Supplement: Supplementary file 2 — Supporting Information S2 [file EJSC-24-1883-s002.docx]

**Appendix 2**

1. **Additional information regarding quantitative methods**

***Incidence calculations***

The overall rate of concussions per 1000 athletic exposures (AEs) was calculated by summing the number of concussions (overall and logged) divided by the total number of AEs and multiplying by 1000 for each team. One unit of AE was defined as one game or one training session. This rate was also calculated for the CMP-logged concussions:

$$Concussion incidence per 1000 AEs=\sum\frac{{Concussions}^{a}}{Athletic Exposures}*1000$$

Where *a* is the overall or total number of logged concussions

To calculate the rate of concussions per 1000 match-hours we calculated the proportion of players in each team who consented to participate using the following formulas:

$$\left( 1 \right) Normalised players per team=\frac{Number of consented players per team}{Total number of players per team}*15$$

$$\left( 2 \right) Total hours of match play per team=\frac{normalised players per team*{matches}^{b}*80 minutes}{60 minutes}$$

$$\left( 3 \right) Concussion incidence per 1000 match hours=\sum\frac{{Concussions}^{a}}{Total hours of match play per team}*1000$$

Where *a* is the overall number of concussions or the total number of logged concussions; *b* is the total number of matches played in the 2019 season.

***Mapping of CMP touchpoints***

The stand-down period (SDP) differed between the regions. For Hawke’s Bay and North Harbour, the SDP was 23 days for players under 19 (U19) and 21 days for players aged 19 and over (19+). Otago had a reduced SDP for those players with valid baselines. The SDP was 19 days for U19 and 12 days for 19+ if players had completed a baseline, which aligned with current World Rugby guidelines. Conversely, if players had not completed a baseline, they adhered to the same SDP rules as Hawke’s Bay and North Harbour.

1. **Additional information regarding qualitative methods**

***Data collection***

Semi-structured interview questions were developed by the multidisciplinary research team, pilot tested, and revised according to feedback. Interviews were conducted by one of six researchers who had experience with qualitative research techniques. Each interview was conducted with one research assistant present to record field notes. The data collection team consisted of one male and five female researchers who were all involved in the CMP's implementation. Prior to the interviews, skilled qualitative researchers (SW/AC) trained the interviewers to facilitate consistency. Regular meeting were held to discuss the use of probing questions, and paraphrasing to facilitate understanding.

*Interview Schedule*

A brief, generic outline of questions asked is presented below. These questions were adapted according to stakeholder group.

1. How was your overall experience of the concussion initiative we piloted this season with your team?

*Probing: For example, the good, the bad, highlights, things that stand out for you or things that you found hard to deal with, what worked well and what didn’t, Why?*

1. If we could change or improve your overall experience / experience you had with your team, how could we do that?

*Probing: What is needed? Could we have provided more information, better support with the technology, improved communication with docs, teachers, team, improve your experience with the App?*

1. *(Participant was shown the infographic depicting the phases of NZR’s CMP)*^1^
   Using the infographic here we are going to break the steps of the concussion initiative into 5 parts. For each part tell us (1) what went well, (2) what didn’t, and (3) how we could improve:
   1. Pre-season baseline testing
      1. Were you involved in the baseline testing?
      2. What went well? Why?
      3. What didn’t go well? Why?
      4. How could we improve your experience?
   2. Did you have any players that you thought sustained a suspected concussion this season?
      1. Can you walk us through what happened after this?
      2. Was there any information you provided to the player or care givers post-injury?
      3. Were there things that you found really helpful after it happened?
      4. How could we improve your experience?

*Probing: Did you log it on the App, if you didn’t logged it on the App can you tell why, Did you feel supported through this process or were there pressures challenges from other stakeholders,*

- 1. Did you refer your players to a GP after the suspected concussion?
     1. Did you have discussion with your players on how the GP visit went?
     2. Did you feel that your player got all the information they needed from the GP?
     3. How do you think we could we improve this experience for you and your player?

*Probing: If they attended ED right after the injury, did they get any information there? If they attended ED did they also see a GP that following week?*

- 1. After your player saw the doctor for their concussion diagnosis was there anything you did with the player around returning to school / work or their recovery process?
     1. Did you support or provide any information about returning to work/school?
     2. Can you tell me what you did to get them back to rugby after their concussion?
     3. Do you remember when they returned to contact training? Did they complete the full stand down period?
     4. Did you feel supported taking your player through the GRTP and GRTL process?
     5. How could we improve your experience?
  2. Medical clearance visit with GP
     1. How did the player report this GP visit went?
     2. Did you feel your player got all the information they needed from the GP?
     3. Did you receive any communication after the player had been cleared?
     4. How could we improve your experience?

1. How did you feel about returning players to contact training/playing?
   1. Did you feel they were ready, or do you think they needed more or less time out? Why?
2. How did you feel about returning players to school or work?
   1. Did you feel they were ready, or do you think they needed more or less time out? Why?
3. Are you with NZR’s efforts to inform you and the team you work with about concussions?
4. Anything else you would like to share about your experience?

***Rigor and trustworthiness***

A relativist, study-specific approach to rigor and trustworthiness was adopted.^2,3^ The data collection team was involved in the implementation of the CMP and was thus known to the participants. Within this study, and as part of the broader project, it is our experience that existing relationships and rapport between the interviewers and the participants contributed to the depth of data. However, it is possible these relationships may have affected what participants felt comfortable to share during the interview. Therefore, every effort was made to create a comfortable environment that encouraged unrestricted sharing of experiences.

The analysis for this study concentrated on the facilitators found in each phase. Regular team meetings were held during the analysis phase to explore assumptions and evaluate coherence in interpretation. Regarding saturation, we believe that there is always potential for new insights as long as data continues to be collected.^4,5^ Instead, our focus was on gaining context-sensitive insights from participants that we considered to be critical role players and key informants, as well as on a rigorous, iterative analysis process.^4^

**References**

1. Salmon D, Romanchuk J, Murphy I, et al. Infographic. New Zealand Rugby’s concussion management pathway. *Br J Sports Med*. 2020;54(5):298-299. doi:10.1136/bjsports-2019-100950

2. Burke S. Rethinking ‘validity’ and ‘trustworthiness’ in qualitative inquiry. How might we judge the quality of qualitative research in sport and exercise sciences? In: Smith B, Sparkes A, eds. *Routledge Handbook of Qualitative Research in Sport and Exercise*. Routledge; 2016.

3. Smith B, McGannon K. Developing rigor in qualitative research: problems and opportunities within sport and exercise psychology. *International Review of Sport and Exercise Psychology*. 2018;11(1):101-121. doi:10.1080/1750984X.2017.1317357

4. Braun V, Clarke V. To saturate or not to saturate? Questioning data saturation as a useful concept for thematic analysis and sample-size rationales. *Qual Res Sport Exerc Health*. 2019;00(00):1-16. doi:10.1080/2159676X.2019.1704846

5. Low J. A Pragmatic Definition of the Concept of Theoretical Saturation. *Sociol Focus*. 2019;52(2):131-139. doi:10.1080/00380237.2018.1544514
